# Recurrent Cylindroma of the Scalp: A Cytomorphological Evaluation at Fine Needle Aspiration Cytology

**DOI:** 10.7759/cureus.25152

**Published:** 2022-05-19

**Authors:** K Amita, SV Pournami, R Rashmi, KN Kusuma, P Priyadarshini

**Affiliations:** 1 Pathology, Adichunchanagiri Institute of Medical Sciences, Adichunchanagiri University, BG Nagara, Mandya, IND

**Keywords:** cytomorphology, recurrent, scalp, turban tumour, adnexal tumour

## Abstract

Specific diagnosis of adnexal tumours at fine needle aspiration cytology (FNAC) is challenging. In a recurrent scalp tumour, a startling array of lesions ranging from benign to malignant tumours fall into differential diagnosis. Accurate preoperative diagnosis is essential for planning management and to improve clinical outcome. Herein, we report a rare case of recurrent cylindroma of the scalp in a 62-year-old male patient. Cytology smears showed tight clusters of round to oval basaloid cells, few clusters with peripheral palisading. Basement membrane material was seen surrounding the cell clusters. FNAC diagnosis of a benign adnexal tumour, possibly cylindroma, was made. The diagnosis was confirmed at histopathology. We emphasize the decisive role of FNAC in arriving at an accurate diagnosis of a recurrent scalp tumour.

## Introduction

Cylindromas are rare benign adnexal tumours, accounting for 0.7% of all the adnexal tumours [1]. Most cylindromas are sporadic and solitary; multiple cylindromas commonly occur as part of genetically driven syndromes [2]. Clinically, when multiple cylindromas arise in the scalp, the appearance simulates a hat or a turban, making the term “turban tumour” appropriate.

Recurrence in a cylindroma is rare, mainly attributed to incomplete excision. When a recurrent tumour is encountered in the scalp region, a startling array of lesions ranging from benign to malignant tumours fall into differential diagnosis. Accurate preoperative diagnosis is essential for planning management and in improving clinical outcome.

Despite known benefits of accurate preoperative diagnosis, scanty literature exists describing specific cytomorphological findings of individual adnexal tumours, especially cylindroma [3-8].

Hence we report the cytomorphological findings in a rare case of recurrent cylindroma of the scalp and discuss the differential diagnosis in detail.

## Case presentation

A 62-year-old male presented with swelling over the scalp for 10 years duration. The swelling was slow growing, not associated with pain or discharge. Patient did not complain of any other swelling anywhere else on the body. There was no history of weight loss or loss of appetite. The swelling was excised one year after the immediate appearance of the swelling. As per the patient's narration, swelling was removed in toto initially. Currently, he did not have any details of the radiological or histopathological examination performed during the initial excision. The swelling recurred after a duration of three years from the initial excision. The recurrent swelling was also slow growing in nature. A provisional clinical diagnosis of sebaceous cyst or fibroma was made.

**Figure 1:**
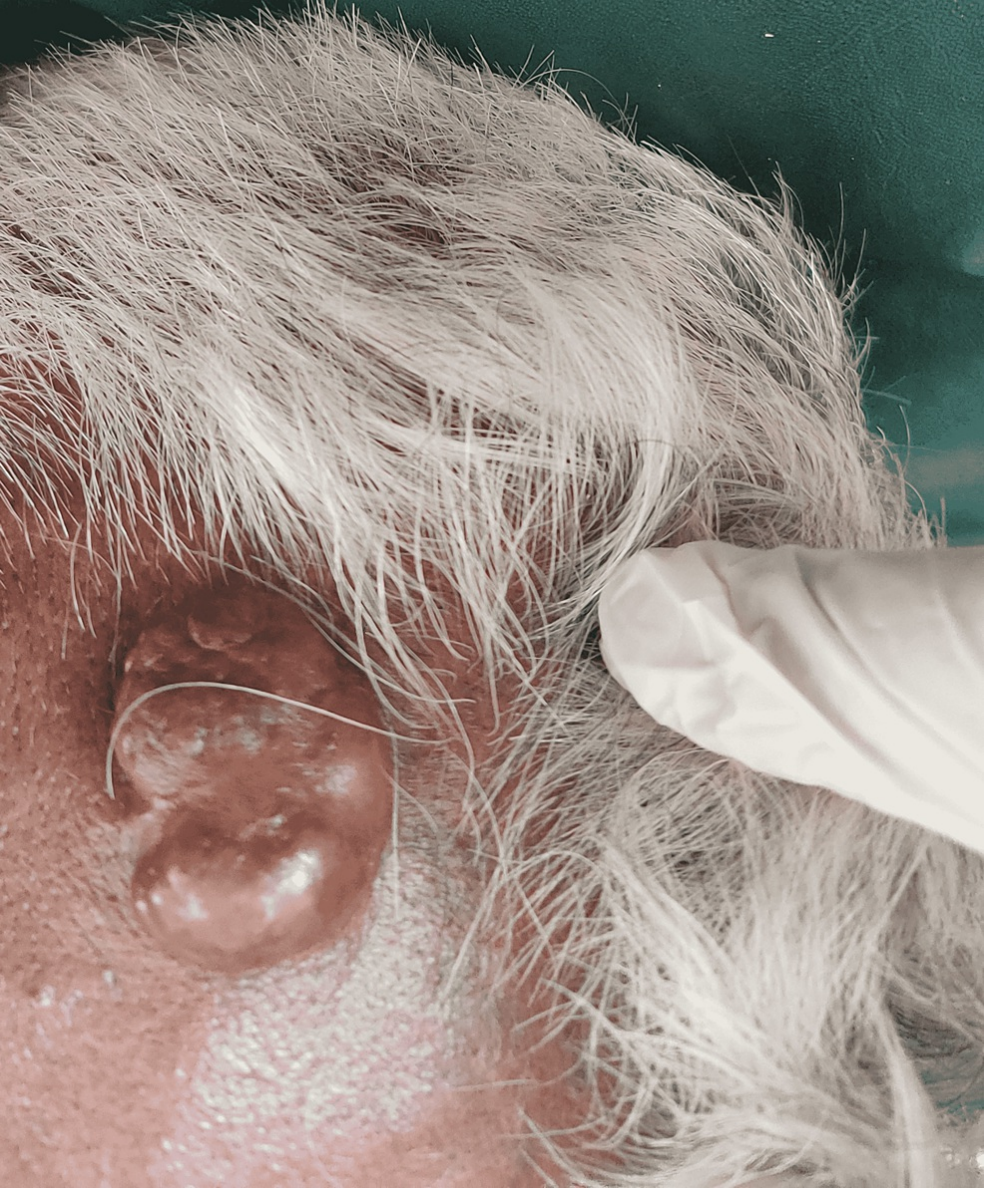
A nodular, bosselated swelling over the frontal region on the scalp.

On examination, swelling was noted in the scalp in the right frontal region. Swelling was nodular, pink, with bosselated appearance, measuring 2.5 x 2 x 2 cm, mobile and non-tender (Figure 1).

It was firm in consistency. The skin overlying the swelling was unremarkable with no evidence of ulceration. On careful examination, no other swelling was identified anywhere else in the body. There was no evidence of regional lymphadenopathy. The patient was well built and well-nourished. Radiological investigations were not performed.

**Figure 2:**
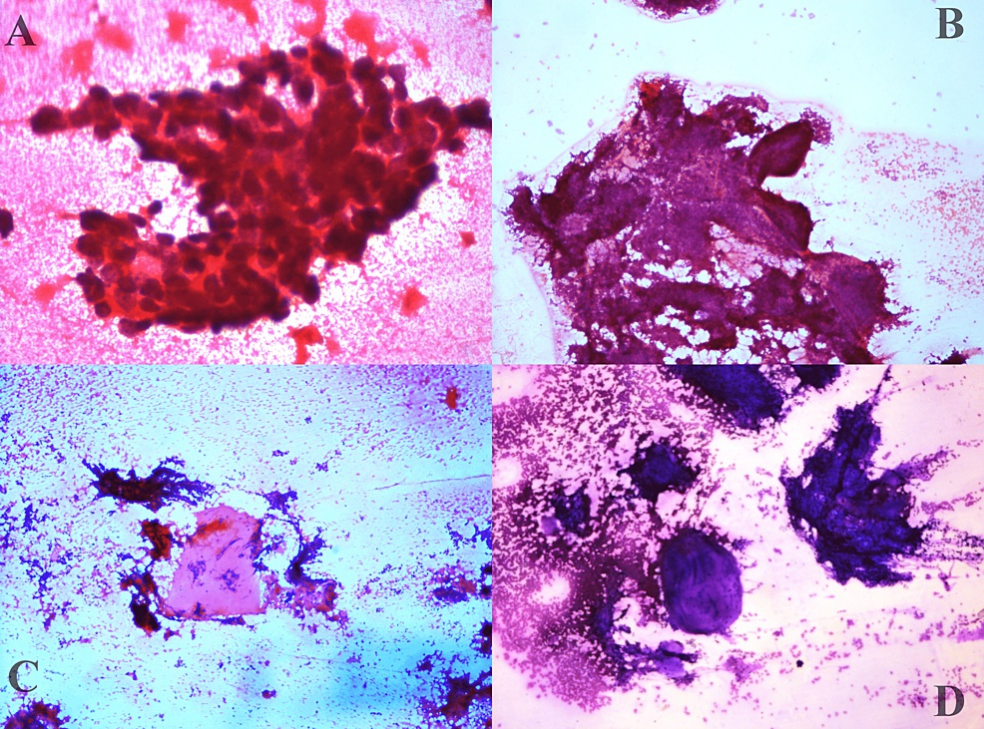
A- fine needle aspiration cytology (FNAC) smears showing basaloid cells. B- peripheral palisading seen in the basaloid cell clusters (Haematoxylin and Eosin, x200). C- basement membrane material (Haematoxylin and Eosin, x100). D- basement membrane material (Giemsa, x200).

**Figure 3:**
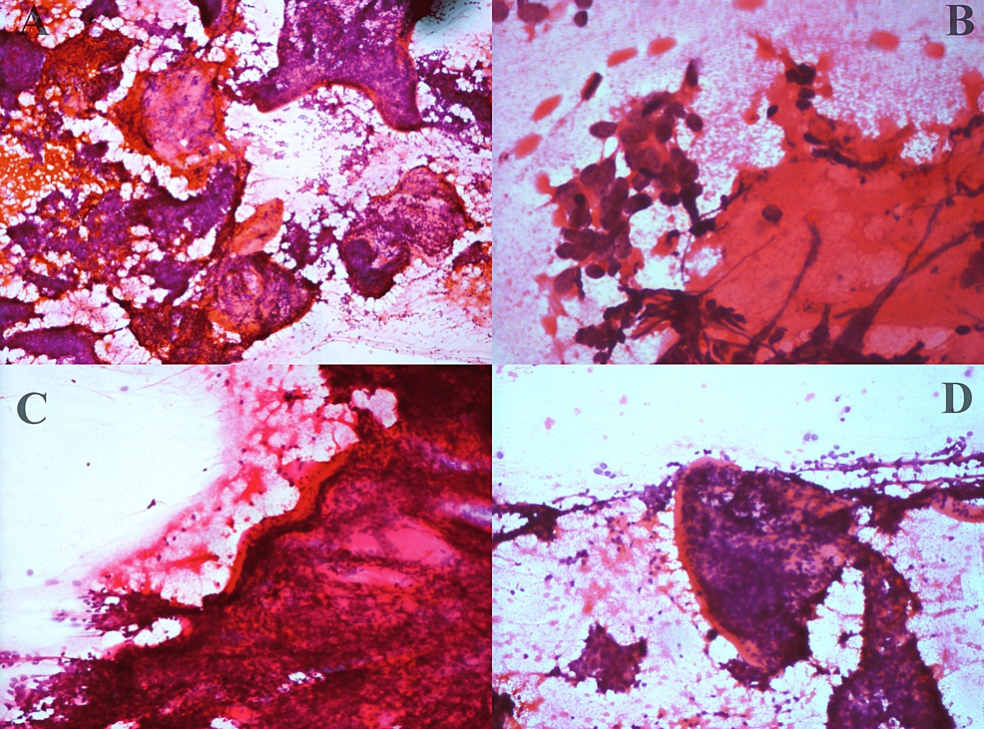
A- fine needle aspiration cytology (FNAC) smears showing basaloid cells along wth basement membrane material (Haematoxylin and Eosin, x100). B- acini adjacent to basement membrane (Haematoxylin and Eosin, x200). C&D- basement membrane enclosing basaloid cells (Haematoxylin and Eosin, x100).

Fine needle aspiration cytology (FNAC) was performed by standard technique, using a 22 gauge needle attached to a Comeco syringe holder. Slides were fixed in 95% ethyl alcohol and stained with haematoxylin and eosin stain. The air-dried slides were stained with Giemsa stain. Smears studied showed moderate cellularity comprising tumour cells in tight sheets, small clusters, vague acini and in singles. In places, some clusters showed peripheral palisading. Individual tumour cells were uniform, small, basaloid, round to oval with dark nuclei and inconspicuous nucleoli. Plenty of thick basement membrane material is seen surrounding the tumour cells and some in the background (Figures 2, 3).

There was no evidence of atypia, mitosis or necrosis in the smears studied. Hence a diagnosis of the benign adnexal tumour with a possibility of cylindroma was made.

The swelling was excised and the specimen was sent for histopathology. On gross examination, the swelling was 2.5 x 2 x 2 cm. The external surface was bosselated. There was no ulceration. The cut surface showed an encapsulated tumour with a solid, uniform, grey-white appearance. There was no evidence of necrosis, haemorrhage or cystic change.

At histopathology, a well-circumscribed, encapsulated tumour was noted. Tumour cells were uniform, basaloid, arranged in jigsaw puzzle pattern. Each cluster exhibited peripheral palisading and was surrounded by thick basement membrane material. Individual tumour cells were round to oval with hyperchromatic nuclei, scanty cytoplasm and inconspicuous nucleoli. There was no evidence of atypia, mitosis, necrosis or haemorrhage. A final diagnosis of benign cylindroma was confirmed at histopathology. There was no evidence of recurrence or any other complaint since eight months of follow-up after the surgical excision of the tumour.

## Discussion

Cyclindromas defy categorization as to eccrine or apocrine origin because of differing evidence of the line of differentiation. Though ultrastructural and immunohistochemical examination point towards the eccrine differentiation, its occurrence in hair follicle-rich regions like the scalp and paucity in eccrine gland-rich areas like the palmoplantar region disputes the eccrine origin theory. The possibility of a hair follicle derivation via epithelial-mesenchymal transition was proposed by Massoumi et al. [9-11].

Cylindromas occur most frequently in the head and neck region with 80% occurring in the scalp area. Sometimes, multiple cylindromas cover a large area on the scalp, giving an appearance of a “turban”, hence the name turban tumour. Other regions where cylindroma has been reported include extremities and trunk with rare cases reported in the orbit, ear, abdomen and breast [7]. These tumours occur in two forms, sporadic and familial. Sporadic cases present as solitary lesions whilst familial cases are associated with multiple tumours of differing differentiation like trichoepithelioma, eccrine spiradenoma which together constitute an autosomal dominant disorder with CYLD gene on chromosome 16q, Brooke Spielger syndrome [2]. One of the important clinical significance of familial forms of cylindroma is their propensity for risk of internal malignancy as well as de novo malignant transformation, making a preoperative accurate diagnosis of cylindroma crucial for further workup and management decisions [12]. This makes FNAC diagnosis very important, in view of easy accessibility and rare reliability on frozen section for definitive diagnosis.

There are few studies describing the role of FNAC in skin adnexal tumours, many restricted to case series. Additionally, one could agree that the subcategorization of benign adnexal tumours at FNAC is largely academic because it rarely makes a difference in clinical management. However, the close relationship of cylindroma to the genetically determined syndrome makes them susceptible to internal malignancy or occurrence of other tumours or tumour-like lesions at other sites, making subcategorization of benign adnexal tumours critically significant. Nevertheless, determining the subcategory will help exclude other malignant primary skin tumours mimicking cylindroma as well as metastatic deposits of look-alikes at FNAC. Finally understanding the differentiation pathway may lead to new inventions with a significant impact on other areas of medicine.

When a tumour that morphologically resembles a cylindroma recurs, differentiating a malignant cylindroma from a benign cylindroma becomes crucial. Similarly, when the true nature of the initial tumour is not known, as was in our case, differentiating it from other skin adnexal tumours like eccrine spiradenoma, trichoepithelioma and other primary malignant or metastatic tumours like adenoid cystic carcinoma and basal cell carcinoma becomes important [3,5,8].

Cylindromas at FNAC show moderate to abundant cellularity with two major constituents. The first is the predominant basaloid cells and the second is the extensive deposits of basement membrane material [3,5]. Other tumours depicting this pattern include adenoid cystic carcinoma (ADCC) and eccrine spiradenoma [3,13,14]. Primary adenoid cystic carcinoma of the scalp, though rare, has been reported in the literature. Additionally, metastasis of ADCC to the scalp region also occurs [15]. Differentiating cylindroma from ADCC on cytology is challenging. Bondeson et al. in their report impressed upon the need to restrict surgical resection before definitive diagnosis, as distinguishing ADCC from cylindroma in the salivary gland is difficult [3]. At times, even at histopathology ADCC may be difficult to distinguish from a cylindroma [13].

It is important to pay careful attention to the overall smear pattern and the details while attempting to distinguish ADCC from cylindroma. The characteristic patterns seen in ADCC, like the cup-shaped structures, three-dimensional cell clusters and many bare nuclei are sparingly seen in cylindroma [16]. Individual tumour cells in ADCC are hyperchromatic with irregular nuclear membrane, demonstrate characteristic nuclear moulding and brisk mitotic activity [6,8]. Similarly, the basement membrane material seen in ADCC is thin and transparent as compared to thick textured basement membrane in cylindroma.

The salient distinguishing features between cylindroma and other entities like eccrine spiradenoma, trichoepithelioma, adenoid cystic carcinoma and basal cell carcinoma are shown in Table 1.

**Table 1:** Distinguishing features of cylindroma from other look-alike lesions at cytology

	Cylindroma	Adenoid cystic carcinoma	Eccrine spiradenoma	Basal cell carcinoma
Pattern	Tight sheets Vague acini Palisading	Acini 3D structures cup-shaped clusters	Sheets Acini Rosettes	Sheets Clusters Palisading
Nuclei	Round to oval, uniform, fine chromatin and inconspicuous nucleoli	Nuclei hyperchromatic, with irregular nuclear membrane, nuclear moulding Brisk mitotic activity	Epithelial and myoepithelial cells Epithelial -Round to oval, uniform, fine chromatin and inconspicuous nucleoli Myoepithelial- Spindle with dark nuclei	Dark, hyperchromatic nuclei Brisk mitotic activity
Basement membrane material	Present, attached to cell clusters Thick	Present, Thin, varying sized globules Tumour cells surrounding the globules	Present Lymphocytes present	Absent
Mucin	Absent	Present	Absent	-
Immunocytochemistry	Epithelial cells -CK 7 Myoepithelial cells- p63, S100	P63, S 100 Ki67 raised	Epithelial cells -CK 7 Myoepithelial cells- p63, S100 Beta catenin and IKH4 positive	EMA,p16,SOX 2

Malignant transformation of cylindroma is rare and may arise in a setting of multiple cylindromatosis. Malignant cylindromas are aggressive tumours with a propensity for local destruction and distant metastasis [12]. In a scalp lesion that has recurred, determining the nature as benign or malignant is crucial for planning management. Though there are no reports of FNAC diagnosis of malignant cylindroma, the presence of atypia, mitosis and necrosis, as would be seen in histopathology would be pointers to the malignant nature at cytology. In our case, none of the above features were present, hence a diagnosis of benign cylindroma was made which was confirmed on histopathology.

## Conclusions

We report a rare case of recurrent cylindroma of scalp diagnosed at cytology. Cylindroma should be considered in the differential diagnosis of basaloid cell rich cytology aspirates from a scalp tumour. The present case underscores the importance of considering cylindroma in differential diagnosis of basaloid cell rich cytology aspirates from a scalp tumour. Careful attention to the pattern and cellular details helps in differentiating it from the look likes like adenoid cystic carcinoma, eccrine spiradenoma and basal cell carcinoma.
